# The genome sequence of the Black Medic, *Medicago lupulina* L.

**DOI:** 10.12688/wellcomeopenres.23134.1

**Published:** 2024-10-09

**Authors:** Markus Ruhsam, Royal Botanic Garden Edinburgh Genome Acquisition Lab, Darwin Tree of Life Barcoding collective, Plant Genome Sizing collective, Wellcome Sanger Institute Tree of Life Management, Samples and Laboratory team, Wellcome Sanger Institute Scientific Operations: Sequencing Operations, Wellcome Sanger Institute Tree of Life Core Informatics team, Tree of Life Core Informatics collective, Darwin Tree of Life Consortium

**Affiliations:** 1 Royal Botanic Garden Edinburgh, Edinburgh, Scotland, UK

**Keywords:** Medicago lupulina, Black Medic, genome sequence, chromosomal, Fabales

## Abstract

We present a genome assembly from a specimen of Black Medic, *Medicago lupulina* (Streptophyta; Magnoliopsida; Fabales; Fabaceae). The genome sequence has a total length of 575.40 megabases. Most of the assembly is scaffolded into 8 chromosomal pseudomolecules. The mitochondrial and plastid genome assemblies have lengths of 294.12 kilobases and 123.99 kilobases, respectively. Gene annotation of this assembly on Ensembl identified 27,424 protein-coding genes.

## Species taxonomy

Eukaryota; Viridiplantae; Streptophyta; Streptophytina; Embryophyta; Tracheophyta; Euphyllophyta; Spermatophyta; Magnoliopsida; Mesangiospermae; eudicotyledons; Gunneridae; Pentapetalae; rosids; fabids; Fabales; Fabaceae; Papilionoideae; 50 kb inversion clade; NPAAA clade; Hologalegina; IRL clade; Trifolieae; *Medicago; Medicago lupulina* L. (NCBI:txid47085).

## Background

**Figure 1.**
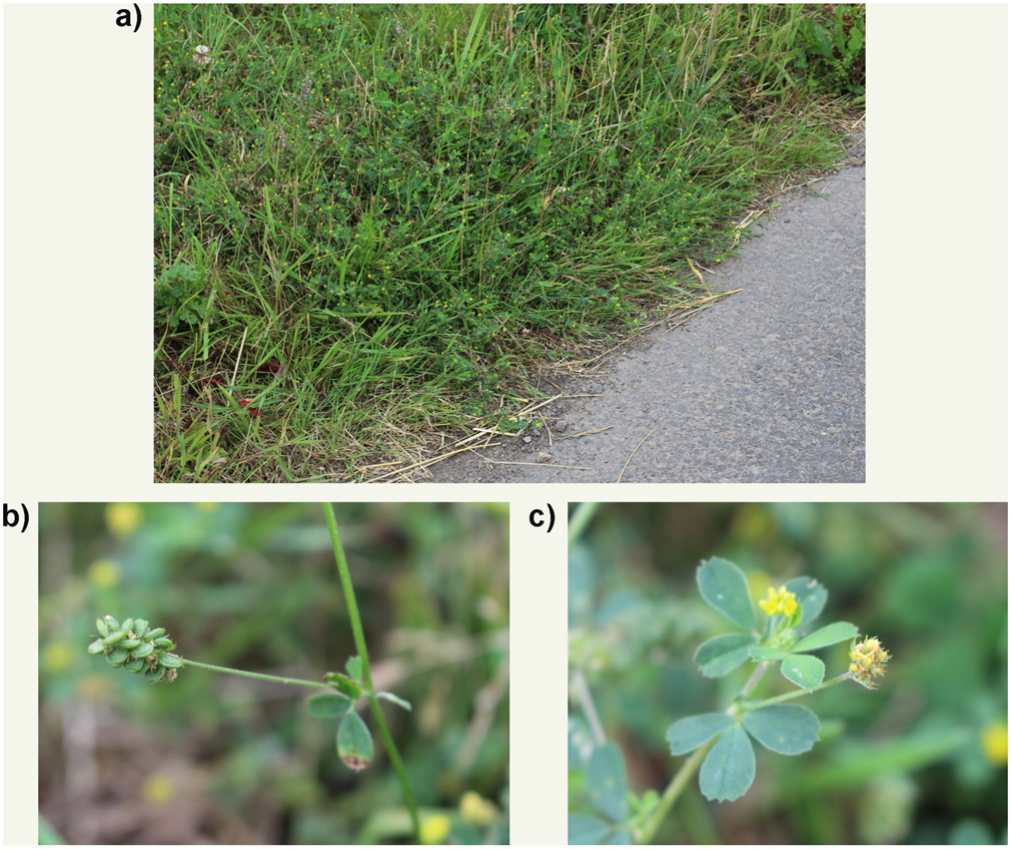
Photographs of the *Medicago lupulina* population ( **a**) from which the sequenced plant was sampled, with details of fruit ( **b**), and flowers and leaves ( **c**).

*Medicago lupulina* (Black Medic) ( Figure 1) is a procumbent to scrambling annual, but may also be a short-lived perennial ( Stace *et al.*, 2019). It is a native of Europe, North Africa and Western and Southern Asia but has now been introduced to large parts of the Americas, Australia, Central and Northern Asia ( POWO, 2024). This species is common in many places and usually associated with habitats influenced by humans occurring in pastures, lawns, pavement cracks and other disturbed areas. It is generally regarded as a weed but has been cultivated in the Old and New World as green fodder for livestock, green manure and as a hay crop ( Turkington & Cavers, 1978).

*Medicago lupulina* has a mixed breeding system with conflicting reports regarding the frequency of insect visitors (summarised in Turkington & Cavers, 1978). Lammerink (1968) found that it can vary from complete self-fertilisation to considerable rates of outcrossing and that there was no difference in seed germination rates between the two modes of fertilisation. The leaf surface of Black Medic is covered by simple and gland-tipped trichomes, which have been shown to deter insect damage such as whitefly oviposition ( Goertzen & Small, 1993).

*Medicago lupulina* has been reported to be a diploid species with 2 *n* = 16 ( Stace *et al.*, 2019). Here we present a chromosomally complete genome sequence for *M. lupulina*, based on a specimen from Gorebridge, Scotland, UK.

## Genome sequence report

The genome size of the *Medicago lupulina* specimen was estimated using flow cytometry, and the (1C-value) was estimated to be 0.75 pg, equivalent to 0.73 Mb. The genome was sequenced using Pacific Biosciences single-molecule HiFi long reads, generating a total of 18.49 Gb (gigabases) from 1.62 million reads, providing approximately 69-fold coverage. Primary assembly contigs were scaffolded with chromosome conformation Hi-C data, which produced 125.59 Gb from 831.75 million reads, yielding an approximate coverage of 218-fold. Specimen and sequencing information is summarised in Table 1.

**Table 1.** Specimen and sequencing data for *Medicago lupulina*.

Project information			
**Study title**	Medicago lupulina		
**Umbrella BioProject**	PRJEB62565		
**Species**	*Medicago lupulina*		
**BioSample**	SAMEA11478582		
**NCBI taxonomy ID**	47085		
Specimen information			
**Technology**	**ToLID**	**BioSample accession**	**Organism part**
**PacBio long read sequencing**	drMedLupu1	SAMEA11478878	leaf
**Hi-C sequencing**	drMedLupu1	SAMEA11478878	leaf
**RNA sequencing**	drMedLupu1	SAMEA11478878	leaf
Sequencing information			
**Platform**	**Run accession**	**Read count**	**Base count (Gb)**
**Hi-C Illumina NovaSeq 6000**	ERR11496084	8.32e+08	125.59
**PacBio Sequel IIe**	ERR11483515	2.75e+06	28.23
**PacBio Sequel IIe**	ERR11483516	1.62e+06	18.49
**RNA Illumina NovaSeq 6000**	ERR11496085	6.12e+07	9.25

**Figure 2.**
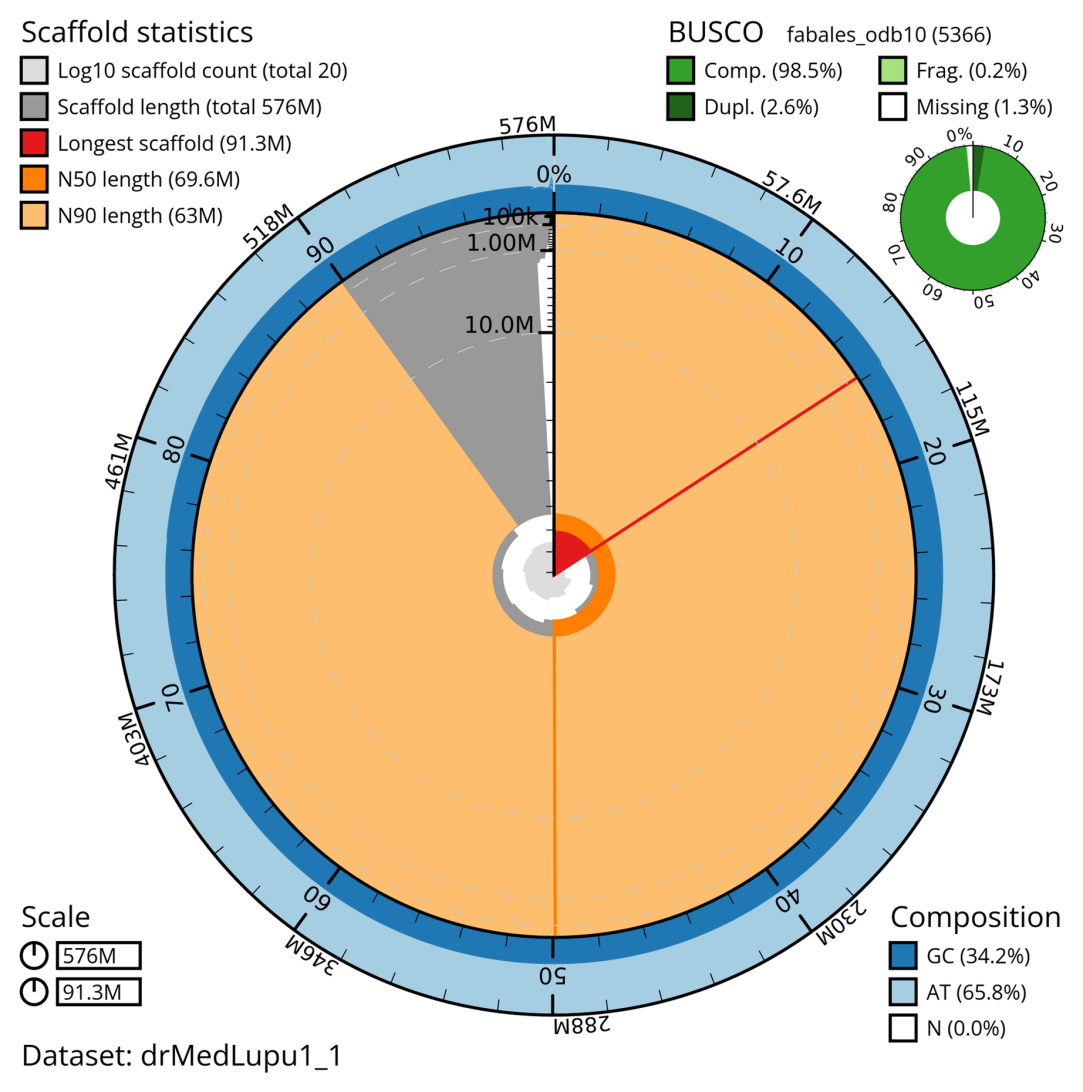
Genome assembly of *Medicago lupulina*, drMedLupu1.1: metrics. The BlobToolKit snail plot shows N50 metrics and BUSCO gene completeness. The main plot is divided into 1,000 size-ordered bins around the circumference with each bin representing 0.1% of the 575,848,570 bp assembly. The distribution of scaffold lengths is shown in dark grey with the plot radius scaled to the longest scaffold present in the assembly (91,320,517 bp, shown in red). Orange and pale-orange arcs show the N50 and N90 scaffold lengths (69,616,069 and 62,961,867 bp), respectively. The pale grey spiral shows the cumulative scaffold count on a log scale with white scale lines showing successive orders of magnitude. The blue and pale-blue area around the outside of the plot shows the distribution of GC, AT and N percentages in the same bins as the inner plot. A summary of complete, fragmented, duplicated and missing BUSCO genes in the fabales_odb10 set is shown in the top right. An interactive version of this figure is available at https://blobtoolkit.genomehubs.org/view/drMedLupu1_1/dataset/drMedLupu1_1/snail.

**Figure 3.**
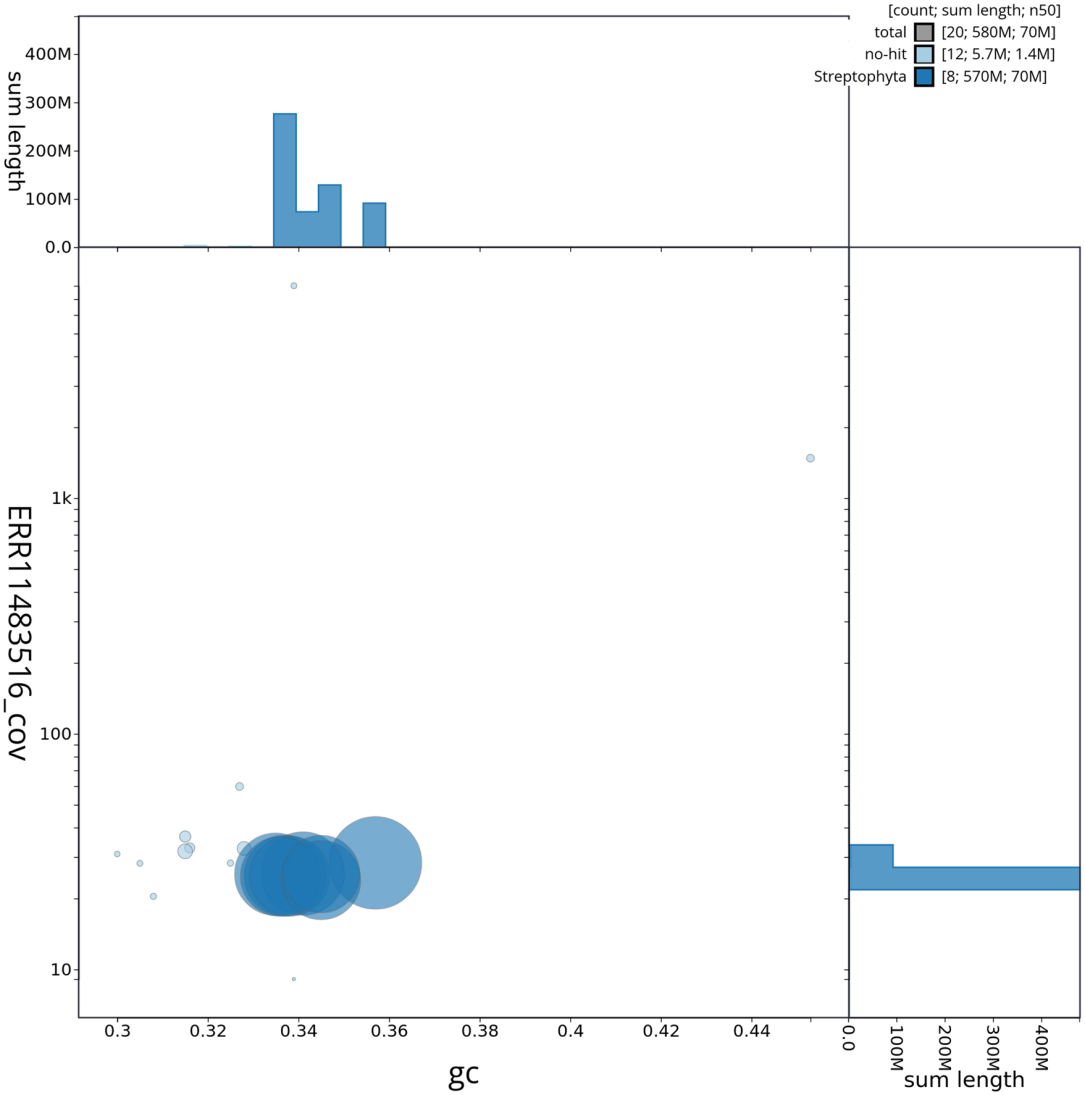
Genome assembly of *Medicago lupulina*, drMedLupu1.1: Blob plot of base coverage in ERR11483516 against GC proportion for sequences in assembly drMedLupu1.1. Sequences are coloured by phylum. Circles are sized in proportion to sequence length. Histograms show the distribution of sequence length sum along each axis. An interactive version of this figure is available at https://blobtoolkit.genomehubs.org/view/drMedLupu1_1/dataset/drMedLupu1_1/blob.

**Figure 4.**
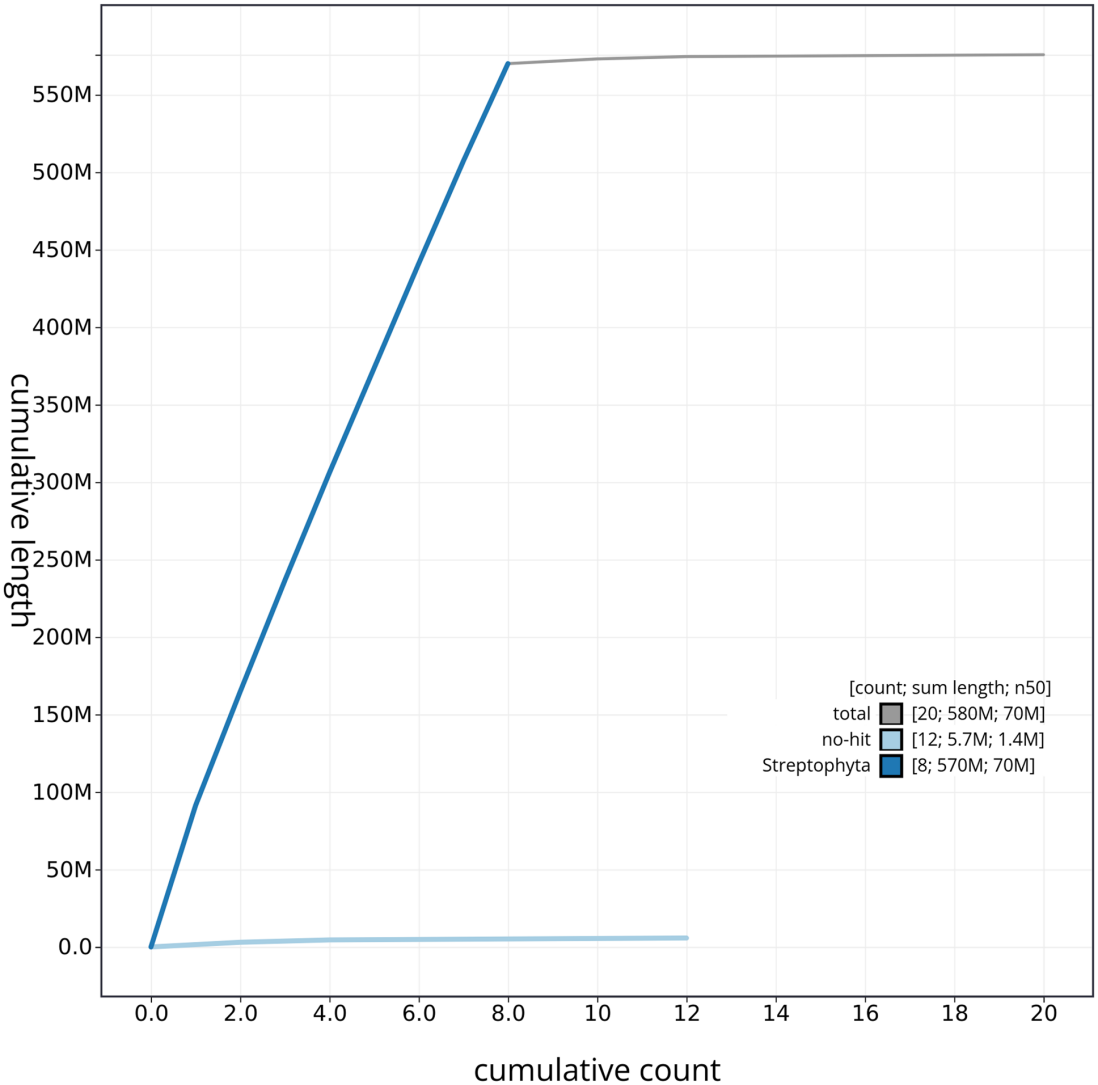
Genome assembly of *Medicago lupulina* drMedLupu1.1: BlobToolKit cumulative sequence plot. The grey line shows cumulative length for all sequences. Coloured lines show cumulative lengths of sequences assigned to each phylum using the buscogenes taxrule. An interactive version of this figure is available at https://blobtoolkit.genomehubs.org/view/drMedLupu1_1/dataset/drMedLupu1_1/cumulative.

**Figure 5.**
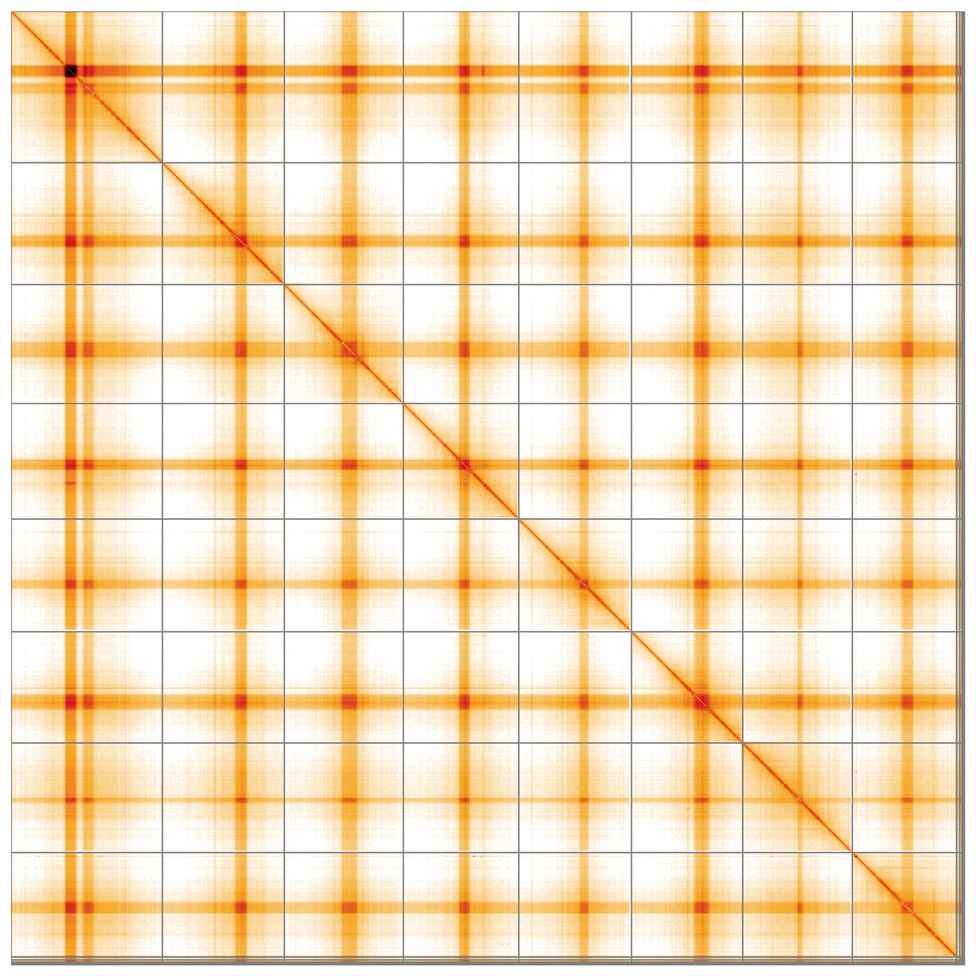
Genome assembly of *Medicago lupulina*, drMedLupu1.1: Hi-C contact map of the drMedLupu1.1 assembly, visualised using HiGlass. Chromosomes are shown in order of size from left to right and top to bottom. An interactive version of this figure may be viewed at https://genome-note-higlass.tol.sanger.ac.uk/l/?d=b8tWZKDRTa6KNdXOBybKMg.

Manual assembly curation corrected 175 missing joins or mis-joins, reducing the scaffold number by 88.02%, and increasing the scaffold N50 by 10.57%. The final assembly has a total length of 575.40 Mb in 18 sequence scaffolds with a scaffold N50 of 69.6 Mb ( Table 2) with 465 gaps. The snail plot in Figure 2 provides a summary of the assembly statistics, while the distribution of assembly scaffolds on GC proportion and coverage is shown in Figure 3. The cumulative assembly plot in Figure 4 shows curves for subsets of scaffolds assigned to different phyla. Most (99%) of the assembly sequence was assigned to 8 chromosomal-level scaffolds. Chromosome-scale scaffolds confirmed by the Hi-C data are named in order of size ( Figure 5; Table 3). The scaffolds are of uncertain order and orientation on chromosome 1 in the region 32–39.5 Mbp. While not fully phased, the assembly deposited is of one haplotype. Contigs corresponding to the second haplotype have also been deposited. The mitochondrial and plastid genomes were also assembled and can be found as contigs within the multifasta file of the genome submission.

**Table 2.** Genome assembly data for *Medicago lupulina*, drMedLupu1.1.

Genome assembly		
Assembly name	drMedLupu1.1	
Assembly accession	GCA_958299785.1	
*Accession of alternate haplotype*	*GCA_958299825.1*	
Span (Mb)	575.40	
Number of contigs	485	
Contig N50 length (Mb)	2.9	
Number of scaffolds	18	
Scaffold N50 length (Mb)	69.6	
Longest scaffold (Mb)	91.32	
Assembly metrics *		*Benchmark*
Consensus quality (QV)	65.6	*≥ 50*
*k*-mer completeness	100.0%	*≥ 95%*
BUSCO **	C:98.5%[S:96.0%,D:2.6%], F:0.2%,M:1.3%,n:5,366	*C ≥ 95%*
Percentage of assembly mapped to chromosomes	99%	*≥ 95%*
Organelles	Mitochondrial genome: 294.12 kb; plastid genome: 123.99 kb	*complete single alleles*
Genome annotation at Ensembl		
Number of protein-coding genes	27,424	
Number of non-coding genes	8,796	
Number of gene transcripts	45,176	

* Assembly metric benchmarks are adapted from column VGP-2020 of “Table 1: Proposed standards and metrics for defining genome assembly quality” from Rhie *et al.* (2021). ** BUSCO scores based on the fabales_odb10 BUSCO set using version 5.4.3. C = complete [S = single copy, D = duplicated], F = fragmented, M = missing, n = number of orthologues in comparison. A full set of BUSCO scores is available at https://blobtoolkit.genomehubs.org/view/drMedLupu1_1/dataset/drMedLupu1_1/busco.

**Table 3.** Chromosomal pseudomolecules in the genome assembly of *Medicago lupulina*, drMedLupu1.

INSDC accession	Name	Length (Mb)	GC%
OY283139.1	1	91.32	35.5
OY283140.1	2	73.45	34.0
OY283141.1	3	71.72	33.5
OY283142.1	4	69.62	33.5
OY283143.1	5	67.91	34.0
OY283144.1	6	67.01	33.5
OY283145.1	7	66.13	34.5
OY283146.1	8	62.96	34.5
OY283147.1	MT	0.29	45.5
OY283148.1	Pltd	0.12	34.0

The estimated Quality Value (QV) of the final assembly is 65.6 with *k*-mer completeness of 100.0%, and the assembly has a BUSCO v5.4.3 completeness of 98.5% (single = 96.0%, duplicated = 2.6%), using the fabales_odb10 reference set ( *n* = 5,366).

Metadata for specimens, BOLD barcode results, spectra estimates, sequencing runs, contaminants and pre-curation assembly statistics are given at https://links.tol.sanger.ac.uk/species/47085.

## Genome annotation report

The *Medicago lupulina* genome assembly (GCA_958299785.1) was annotated at the European Bioinformatics Institute (EBI) on Ensembl Rapid Release. The resulting annotation includes 45,176 transcribed mRNAs from 27,424 protein-coding and 8,796 non-coding genes ( Table 2; https://rapid.ensembl.org/Medicago_lupulina_GCA_958299785.1/Info/Index). The average transcript length is 3,395.57. There are 1.25 coding transcripts per gene and 4.85 exons per transcript.

## Methods

### Sample acquisition, DNA barcoding and genome size estimation

Leaves of *Medicago lupulina* (specimen ID EDTOL02227, ToLID drMedLupu1) were collected from Gorebridge, Scotland, UK (latitude 55.85, longitude –3.03) on 2021-07-30. The specimen was collected and identified by Markus Ruhsam (Royal Botanic Garden Edinburgh) and preserved by liquid nitrogen. The herbarium voucher associated with the sequenced plant ( https://data.rbge.org.uk/herb/E01152298) was deposited in the herbarium of RBG Edinburgh (E).

The initial species identification was verified by an additional DNA barcoding process according to the framework developed by Twyford *et al*. (2024). Part of the plant specimen was preserved in silica gel desiccant. A DNA extraction from the dried plant was amplified by PCR for standard barcode markers, with the amplicons sequenced and compared to public sequence databases including GenBank and the Barcode of Life Database (BOLD). The barcode sequences for this specimen are openly available on BOLD ( Ratnasingham & Hebert, 2007). Following whole genome sequence generation, DNA barcodes were also used alongside the initial barcoding data for sample tracking through the genome production pipeline at the Wellcome Sanger Institute ( Twyford *et al.*, 2024). The standard operating procedures for the Darwin Tree of Life barcoding have been deposited on protocols.io ( Beasley *et al.*, 2023).

The genome size was estimated by flow cytometry using the fluorochrome propidium iodide and following the ‘one-step’ method as outlined in Pellicer *et al.* (2021). For this species, the General Purpose Buffer (GPB) supplemented with 3% PVP and 0.08% (v/v) beta-mercaptoethanol was used for isolation of nuclei ( Loureiro *et al.*, 2007), and the internal calibration standard was *Petroselinum crispum* ‘Champion Moss Curled’ with an assumed 1C-value of 2,200 Mb ( Obermayer *et al.*, 2002).

### Nucleic acid extraction

The workflow for high molecular weight (HMW) DNA extraction at the WSI Tree of Life Core Laboratory includes a sequence of core procedures: sample preparation and homogenisation, DNA extraction, fragmentation and purification. Detailed protocols are available on protocols.io ( Denton *et al.*, 2023). The drMedLupu1 sample was weighed and dissected on dry ice ( Jay *et al.*, 2023) and leaf tissue was cryogenically disrupted using the Covaris cryoPREP ^®^ Automated Dry Pulverizer ( Narváez-Gómez *et al.*, 2023). HMW DNA was extracted using the Automated Plant MagAttract v2 protocol ( Todorovic *et al.*, 2023). HMW DNA was sheared into an average fragment size of 12–20 kb in a Megaruptor 3 system ( Bates *et al.*, 2023). Sheared DNA was purified by solid-phase reversible immobilisation, using AMPure PB beads to eliminate shorter fragments and concentrate the DNA ( Strickland *et al.*, 2023). The concentration of the sheared and purified DNA was assessed using a Nanodrop spectrophotometer and Qubit Fluorometer and Qubit dsDNA High Sensitivity Assay kit. Fragment size distribution was evaluated by running the sample on the FemtoPulse system.

RNA was extracted from leaf tissue of drMedLupu1 in the Tree of Life Laboratory at the WSI using the RNA Extraction: Automated MagMax™ *mir*Vana protocol ( do Amaral *et al.*, 2023). The RNA concentration was assessed using a Nanodrop spectrophotometer and a Qubit Fluorometer using the Qubit RNA Broad-Range Assay kit. Analysis of the integrity of the RNA was done using the Agilent RNA 6000 Pico Kit and Eukaryotic Total RNA assay.

### Library preparation and nucleic acid sequencing

Library preparation and DNA sequencing were performed at the WSI Scientific Operations core. Pacific Biosciences HiFi circular consensus DNA sequencing libraries were prepared using the PacBio Express Template Preparation Kit v2.0 (Pacific Biosciences, California, USA) as per the manufacturer's instructions. The kit includes the reagents required for removal of single-strand overhangs, DNA damage repair, end repair/A-tailing, adapter ligation, and nuclease treatment. Library preparation also included a library purification step using 0.8X AMPure PB beads (Pacific Biosciences, California, USA) and size selection step to remove templates <5 kb using AMPure PB modified SPRI. DNA concentration was quantified using the Qubit Fluorometer v2.0 and Qubit HS Assay Kit and the final library fragment size analysis was carried out using the Agilent Femto Pulse Automated Pulsed Field CE Instrument and gDNA 55 kb BAC analysis kit.

Poly(A) RNA-Seq libraries were constructed using the NEB Ultra II RNA Library Prep kit and RNA sequencing was performed on an Illumina NovaSeq 6000 instrument.

Samples were sequenced using the Sequel IIe system (Pacific Biosciences, California, USA). The concentration of the library loaded onto the Sequel IIe was within the manufacturer's recommended loading concentration range of 40–100 pM. The SMRT link software, a PacBio web-based end-to-end workflow manager, was used to set-up and monitor the run, as well as perform primary and secondary analysis of the data upon completion.

### Hi-C Crosslinking, library preparation, and sequencing

Hi-C data was generated at the WSI Scientific Operations core from leaf tissue of drMedLupu1, using the Arima-HiC v2 kit. Tissue was finely ground using cryoPREP and then subjected to nuclei isolation using a modified protocol of the Qiagen QProteome Kit. After isolation, the nuclei were fixed, and the DNA crosslinked using 37% formaldehyde solution. The crosslinked DNA was then digested using the restriction enzyme master mix. The 5’-overhangs were then filled in and labelled with biotinylated nucleotides and proximally ligated. An overnight incubation was carried out for enzymes to digest remaining proteins and for crosslinks to reverse. A clean up is performed with SPRIselect beads prior to library preparation. DNA concentration was quantified using the Qubit Fluorometer v2.0 and Qubit HS Assay Kit according to the manufacturer’s instructions. For Hi-C library preparation, DNA was fragmented to a size of 400 to 600 bp using a Covaris E220 sonicator. The DNA was then enriched, barcoded, and amplified using the NEBNext Ultra II DNA Library Prep Kit, following manufacturers’ instructions. The Hi-C sequencing was performed using paired-end sequencing with a read length of 150 bp on an Illumina NovaSeq 6000 instrument.

### Genome assembly, curation and evaluation

***Assembly***

The HiFi reads were first assembled using Hifiasm ( Cheng *et al.*, 2021) with the --primary option. Haplotypic duplications were identified and removed using purge_dups ( Guan *et al.*, 2020). The Hi-C reads were mapped to the primary contigs using bwa-mem2 ( Vasimuddin *et al.*, 2019). The contigs were further scaffolded using the provided Hi-C data ( Rao *et al.*, 2014) in YaHS ( Zhou *et al.*, 2023) using the --break option. The scaffolded assemblies were evaluated using Gfastats ( Formenti *et al.*, 2022), BUSCO ( Manni *et al.*, 2021) and MERQURY.FK ( Rhie *et al.*, 2020). The organelle genomes were assembled using MitoHiFi ( Uliano-Silva *et al.*, 2023) and OATK ( Zhou, 2023).

***Curation***

The assembly was decontaminated using the Assembly Screen for Cobionts and Contaminants (ASCC) pipeline (article in preparation). Manual curation was primarily conducted using PretextView ( Harry, 2022), with additional insights provided by JBrowse2 ( Diesh *et al.*, 2023) and HiGlass ( Kerpedjiev *et al.*, 2018). Scaffolds were visually inspected and corrected as described by Howe *et al*. (2021). Any identified contamination, missed joins, and mis-joins were corrected, and duplicate sequences were tagged and removed. The process is documented at https://gitlab.com/wtsi-grit/rapid-curation (article in preparation).

***Evaluation of final assembly***

A Hi-C map for the final assembly was produced using bwa-mem2 ( Vasimuddin *et al.*, 2019) in the Cooler file format ( Abdennur & Mirny, 2020). To assess the assembly metrics, the *k*-mer completeness and QV consensus quality values were calculated in Merqury ( Rhie *et al.*, 2020). This work was done using the “sanger-tol/readmapping” ( Surana *et al.*, 2023a) and “sanger-tol/genomenote” ( Surana *et al.*, 2023b) pipelines. The genome readmapping pipelines were developed using the nf-core tooling ( Ewels *et al.*, 2020), use MultiQC ( Ewels *et al.*, 2016), and make extensive use of the Conda package manager, the Bioconda initiative ( Grüning *et al.*, 2018), the Biocontainers infrastructure ( da Veiga Leprevost *et al.*, 2017), and the Docker ( Merkel, 2014) and Singularity ( Kurtzer *et al.*, 2017) containerisation solutions. The genome was analysed within the BlobToolKit environment ( Challis *et al.*, 2020) and BUSCO scores ( Manni *et al.*, 2021) were calculated.

Table 4 contains a list of relevant software tool versions and sources.

**Table 4.** Software tools: versions and sources.

Software tool	Version	Source
BlobToolKit	4.2.1	https://github.com/blobtoolkit/blobtoolkit
BUSCO	5.3.2	https://gitlab.com/ezlab/busco
bwa-mem2	2.2.1	https://github.com/bwa-mem2/bwa-mem2
Cooler	0.8.11	https://github.com/open2c/cooler
Gfastats	1.3.6	https://github.com/vgl-hub/gfastats
Hifiasm	0.16.1-r375	https://github.com/chhylp123/hifiasm
HiGlass	1.11.6	https://github.com/higlass/higlass
Merqury	MerquryFK	https://github.com/thegenemyers/MERQURY.FK
MitoHiFi	3	https://github.com/marcelauliano/MitoHiFi
OATK	0.1	https://github.com/c-zhou/oatk
PretextView	0.2	https://github.com/wtsi-hpag/PretextView
purge_dups	1.2.5	https://github.com/dfguan/purge_dups
sanger-tol/ genomenote	v1.0	https://github.com/sanger-tol/genomenote
sanger-tol/ readmapping	1.1.0	https://github.com/sanger-tol/readmapping/tree/1.1.0
YaHS	1.2a.2	https://github.com/c-zhou/yahs

### Genome annotation

The Ensembl Genebuild annotation system ( Aken *et al.*, 2016) was used to generate annotation for the *Medicago lupulina* assembly (GCA_958299785.1) in Ensembl Rapid Release at the EBI. Annotation was created primarily through alignment of transcriptomic data to the genome, with gap filling via protein-to-genome alignments of a select set of proteins from UniProt ( UniProt Consortium, 2019).

### Wellcome Sanger Institute – Legal and Governance

The materials that have contributed to this genome note have been supplied by a Darwin Tree of Life Partner. The submission of materials by a Darwin Tree of Life Partner is subject to the **‘Darwin Tree of Life Project Sampling Code of Practice’**, which can be found in full on the Darwin Tree of Life website here. By agreeing with and signing up to the Sampling Code of Practice, the Darwin Tree of Life Partner agrees they will meet the legal and ethical requirements and standards set out within this document in respect of all samples acquired for, and supplied to, the Darwin Tree of Life Project.

Further, the Wellcome Sanger Institute employs a process whereby due diligence is carried out proportionate to the nature of the materials themselves, and the circumstances under which they have been/are to be collected and provided for use. The purpose of this is to address and mitigate any potential legal and/or ethical implications of receipt and use of the materials as part of the research project, and to ensure that in doing so we align with best practice wherever possible. The overarching areas of consideration are:

•     Ethical review of provenance and sourcing of the material

•     Legality of collection, transfer and use (national and international)

Each transfer of samples is further undertaken according to a Research Collaboration Agreement or Material Transfer Agreement entered into by the Darwin Tree of Life Partner, Genome Research Limited (operating as the Wellcome Sanger Institute), and in some circumstances other Darwin Tree of Life collaborators.

## Data Availability

European Nucleotide Archive: *Medicago lupulina*. Accession number PRJEB62565; https://identifiers.org/ena.embl/PRJEB62565 ( Wellcome Sanger Institute, 2023). The genome sequence is released openly for reuse. The *Medicago lupulina* genome sequencing initiative is part of the Darwin Tree of Life (DToL) project. All raw sequence data and the assembly have been deposited in INSDC databases. Raw data and assembly accession identifiers are reported in Table 1.

## Author information

Members of the Royal Botanic Garden Edinburgh Genome Acquisition Lab are listed here: https://doi.org/10.5281/zenodo.4786682.

Members of the Plant Genome Sizing collective are listed here: https://doi.org/10.5281/zenodo.7994306.

Members of the Darwin Tree of Life Barcoding collective are listed here: https://doi.org/10.5281/zenodo.12158331

Members of the Wellcome Sanger Institute Tree of Life Management, Samples and Laboratory team are listed here: https://doi.org/10.5281/zenodo.12162482.

Members of Wellcome Sanger Institute Scientific Operations: Sequencing Operations are listed here: https://doi.org/10.5281/zenodo.12165051.

Members of the Wellcome Sanger Institute Tree of Life Core Informatics team are listed here: https://doi.org/10.5281/zenodo.12160324.

Members of the Tree of Life Core Informatics collective are listed here: https://doi.org/10.5281/zenodo.12205391.

Members of the Darwin Tree of Life Consortium are listed here: https://doi.org/10.5281/zenodo.4783558.
