# Supplementary material for: The genome sequence of the Black Medic, Medicago lupulina L
Source: Wellcome Open Res. 2024 Nov 4;9:574. Originally published 2024 Oct 9. [Version 2] doi: 10.12688/wellcomeopenres.23134.2 (PMC11503012; doi:10.12688/wellcomeopenres.23134.2)
Supplement: Peer review history

# Reviewer report: Reviewer response for version 2

**DOI:** 10.21956/wellcomeopenres.25756.r109658

**Published:** 2025-09-30

**Authors:** Xin Liu

**Affiliations:** 1 Beijing Genomics Institute, Beijing, China

This is a typical data note article, following a relatively standardized workflow that includes sample collection, specimen preparation and preservation, data generation, genome assembly and annotation, and genome evaluation. Overall, the methods and results are clearly described, with no major issues. I have a few suggestions/comments for consideration:

 1. Compared to the genome size estimated by flow cytometry (730 Mb), the final assembled genome is considerably smaller (575.4 Mb). Although the authors used standard metrics such as k-mer completeness and BUSCO to assess assembly integrity, the substantial discrepancy between the estimated and assembled genome sizes warrants at least a brief discussion in the manuscript, even if a k-mer-based genome size estimation is not performed.

 2. The manuscript states that RNA sequencing was performed using leaf tissue, yet the gene annotation section specifically mentions the number of transcripts. It seems unlikely that this transcript count is fully supported by RNA data. Using RNA-seq from a single tissue (likely at a single time point) to assist genome annotation is not ideal. While this series of genome studies follows a near-standardized pipeline, the annotation process—particularly the extent to which annotated genes are supported by RNA evidence—should be more clearly explained. Otherwise, it may lead to the misunderstanding that transcriptome-wide annotation was achieved using RNA data from just one tissue.

 3. A minor issue: in the first paragraph of the “Genome sequence report” section, the authors describe the amount of sequencing data and mention approximate coverage. This coverage is presumably calculated based on the genome size, but it is unclear which genome size was used for the estimation. It does not appear to be the flow cytometry-based estimate mentioned earlier in the same section.

Are sufficient details of methods and materials provided to allow replication by others?

Yes

Is the rationale for creating the dataset(s) clearly described?

Yes

Are the datasets clearly presented in a useable and accessible format?

Yes

Are the protocols appropriate and is the work technically sound?

Yes

Reviewer Expertise:

plant genomics

I confirm that I have read this submission and believe that I have an appropriate level of expertise to confirm that it is of an acceptable scientific standard.

# Reviewer report: Reviewer response for version 2

**DOI:** 10.21956/wellcomeopenres.25756.r116740

**Published:** 2025-01-16

**Authors:** Harpreet Kaur

**Affiliations:** 1 University of Minnesota, St. Paul, Minnesota, USA

The manuscript titled “The Genome Sequence of the Black Medic, *Medicago lupulina L.*” presents a well-executed genome sequencing, assembly, and annotation of a legume species. The research holds significant value for the community working on legumes and related species, offering a detailed and replicable methodology.

 I have one suggestion for consideration. The estimated genome size based on flow cytometry is reported to be 730 MB, while the assembled genome size is 575.40 MB with 465 gaps. It would be helpful if the authors could provide the estimated genome size using k-mer analysis as well.

 Additionally, an evaluation of the number and proportion of repetitive sequences captured in the assembly could provide further insights into the completeness and quality of the genome assembly. These analyses would enhance the robustness of the manuscript.

Are sufficient details of methods and materials provided to allow replication by others?

Yes

Is the rationale for creating the dataset(s) clearly described?

Yes

Are the datasets clearly presented in a useable and accessible format?

Yes

Are the protocols appropriate and is the work technically sound?

Yes

Reviewer Expertise:

Alfalfa Genetics and Genomics

I confirm that I have read this submission and believe that I have an appropriate level of expertise to confirm that it is of an acceptable scientific standard.

# Reviewer report: Reviewer response for version 2

**DOI:** 10.21956/wellcomeopenres.25756.r109824

**Published:** 2024-12-17

**Authors:** Zhe-Chen Qi

**Affiliations:** 1 College of Life Sciences and Medicine, Zhejiang Province Key Laboratory of Plant Secondary Metabolism and Regulation, Zhejiang Sci-Tech University, Hangzhou, China

The manuscript provides a comprehensive and detailed report on the genome assembly of *Medicago lupulina*, a species of significant interest in plant genomics, particularly in the context of Fabaceae and legume crop improvement. The study is well-structured, presenting clear insights into genome size estimation, sequencing methods, assembly quality, and annotation processes.

Are sufficient details of methods and materials provided to allow replication by others?

Yes

Is the rationale for creating the dataset(s) clearly described?

Yes

Are the datasets clearly presented in a useable and accessible format?

Yes

Are the protocols appropriate and is the work technically sound?

Yes

Reviewer Expertise:

Plant comparative genomics

I confirm that I have read this submission and believe that I have an appropriate level of expertise to confirm that it is of an acceptable scientific standard.

# Reviewer report: Reviewer response for version 1

**DOI:** 10.21956/wellcomeopenres.25474.r105919

**Published:** 2024-10-24

**Authors:** Hoang Dang Khoa Do

**Affiliations:** 1 Nguyen Tat Thanh University, Ho Chi Minh City, Vietnam

The study reported the genomic data of *Medicago lupulina*, including eight chromosomal pseudomolecules, a complete chloroplast genome, and a full mitochondrial genome. Additionally, gene contents were annotated for these genomes and submitted to the public database.

- ** a.** Is the rationale for creating the dataset(s) clearly described?
  The processes for sampling, DNA extraction, sequencing, and analysis were described clearly. Other scientists can repeat the process with the raw data.
- **b. **Are the protocols appropriate and is the work technically sound?
  The protocol for obtaining the genomic data of *Medicago lupulina* was appropriate, which included HiFi long reads, Hi-C, and RNA data. This study provided the genomic features of *Medicago lupulina* which are necessary for further studies about the evolution of this species and its relatives.
- **c.** Are sufficient details of methods and materials provided to allow replication by others?
  The methods and materials were clearly described but it requires experienced scientists to replicate this study as well as the publication of Cobionts and Contaminants (ASCC) pipeline (In preparation).
- **d.** Are the datasets clearly presented in a useable and accessible format?
  The datasets were available online so they could be reused.

The manuscript was well-prepared and clearly presented the results.

 I do not have comments about the current form of the manuscript which is ready for indexing.

 Best regards.

Are sufficient details of methods and materials provided to allow replication by others?

Yes

Is the rationale for creating the dataset(s) clearly described?

Yes

Are the datasets clearly presented in a useable and accessible format?

Yes

Are the protocols appropriate and is the work technically sound?

Yes

Reviewer Expertise:

Plant genomics, Plant systematics, Next-generation sequencing.

I confirm that I have read this submission and believe that I have an appropriate level of expertise to confirm that it is of an acceptable scientific standard.

# Reviewer report: Reviewer response for version 1

**DOI:** 10.21956/wellcomeopenres.25474.r104652

**Published:** 2024-10-22

**Authors:** Xin Liu

**Affiliations:** 1 Beijing Genomics Institute, Beijing, China

This data note reported the genome assembly and gene annotation of Black Medic. The methods used are commonly used and widely accepted for plant genome assembly and analysis. And the final genome assembly seems to be of high quality. So, for this data note, I do not have fundamental questions, and I would recommend for indexing. I have some questions related to some details, which I think should be errors in the description and should be revised.

 In the first paragraph of the 'Genome sequence report', the authors mentioned about the C value and the genome size estimated based on the C value. But I think it should be wrong, as the C value of 0.75 pg., should be equivalent to 0.73 Gb, which should be more reasonable. Also, in this paragraph, the authors mentioned about the data generation and fold coverage information. Again, I think there might be some errors, as it is different from the information mentioned in Table 1 (more PacBio data mentioned in Table 1), and more importantly, the fold coverage estimation seems to be quite odd (for a genome size of 0.73 Gb, or 0.58 Gb, 18.49 Gb seems not to reach 69-fold). It should be mistakes.

 I would suggest the authors to check throughout to avoid that kind of errors, making the data note to be more accurate.

Are sufficient details of methods and materials provided to allow replication by others?

Yes

Is the rationale for creating the dataset(s) clearly described?

Yes

Are the datasets clearly presented in a useable and accessible format?

Yes

Are the protocols appropriate and is the work technically sound?

Yes

Reviewer Expertise:

plant genomics

I confirm that I have read this submission and believe that I have an appropriate level of expertise to confirm that it is of an acceptable scientific standard.
